# Parameter Identification and Transition Process Online Calibration Method of Pulsed Eddy Current Receiving Coil Based on Underdamped Dynamic Response Characteristics

**DOI:** 10.3390/s25134049

**Published:** 2025-06-29

**Authors:** Zhiwu Zeng, Jie Wang, Xiaoju Huang, Yun Zuo, Yuan Liu, Xu Tian, Feng Pei, Kui Liu, Fu Chen, Xiaotian Wang, Jingang Wang

**Affiliations:** 1 Construction Branch State Grid Jiangxi Electric Power Co., Ltd., Nanchang 330036, China; zwzeng21@163.com (Z.Z.); wangjie0513@proton.me (J.W.); huangxiaoju@proton.me (X.H.); yz4547339@sina.com (Y.Z.); ly781015@proton.me (Y.L.); 2 State Grid Jiangxi Electric Power Co., Ltd. Research Institute, Nanchang 330096, China; xt6452319@proton.me (X.T.); pf16548@proton.me (F.P.); 3 Jiangxi Transmission & Substation Engineering Co., Ltd., Nanchang 330077, China; liukui_0226@proton.me (K.L.); cf1651489@proton.me (F.C.); 4 State Key Laboratory of Power Transmission Equipment Technology, School of Electrical Engineering, Chongqing University, Chongqing 400044, China; wxt1834154643@126.com

**Keywords:** underdamped oscillation, real-time calibration, recursive identification, pulsed eddy current

## Abstract

In order to solve the problem that the system parameters will be offset during the detection process of the pulsed eddy current receiving coil, this paper first analyzes the response signal of the receiving system and the deconvolution process of the response signal, and discusses the influence of various system parameters on the deconvolution accuracy. A method is proposed to change the system response characteristics and apply a step signal through the excitation coil to realize parameter identification through the response of the receiving coil system. The error of feature extraction under the change in each parameter is discussed, and the influence of increasing the matching resistance and switching the capacitor in parallel on the identification accuracy is analyzed and compared. It is proposed to realize the accurate identification of the receiving system through the Newton method. It is proved from both simulation and experiment that the method proposed in this paper can realize the identification of the receiving coil parameters efficiently, conveniently, and accurately, and can improve the inversion accuracy of the pulsed eddy current detection signal and improve the detection accuracy.

## 1. Introduction

The small coil time-domain pulsed eddy current method has been widely used in geological exploration in the fields of mining, transportation, water conservancy, and urban underground space due to its advantages of convenient construction, rapidity, and sensitivity to low-resistance objects [1,2,3]. This method generally consists of an excitation coil and a receiving coil. The excitation coil emits a pulse current to generate eddy currents in the terrain object to be detected. The receiving coil senses and receives the eddy current signal of the object to be detected. The time-domain pulsed eddy current method realizes the detection of objects in different strata by analyzing the received signals at different times [4,5].

In the process of sensing the eddy current signal of the object to be detected, due to the parameters of the receiving coil itself, the collected signal is equivalent to passing through a transmission system [6,7,8]. Affected by the transmission system, the collected signal will be distorted compared with the induced signal. In order to obtain the induced magnetic field change, it is necessary to perform a deconvolution transformation on the collected signal to remove the influence of the receiving coil system. This process is called the transition process of the receiving coil [9]. In order to eliminate the influence of the transition process, it is necessary to perform a deconvolution transformation on the collected signal based on the known transfer function of the receiving coil system and the collected induced voltage so as to realize the calibration of the collected signal [10]. The calibration accuracy of the signal is affected by the parameters of the receiving coil transfer function, so it is very important to obtain an accurate system transfer function [11].

In order to accurately measure the secondary field response through the distorted receiving coil output signal, a mapping between the coil-induced electromotive force and the output signal is established [12]. For coil sensors, the commonly used calibration method is to establish a controllable calibration magnetic field in space and solve the coil calibration file by analyzing the relationship between the coil-induced electromotive force and its output signal [13]. Usually, a sinusoidal signal is used as the calibration signal. Several frequency values are selected within the frequency range under investigation. The amplitude and phase angle of the input and steady-state output signals at each calibration frequency are measured respectively. The transfer function of the coil to be tested is obtained by fitting the experimental data. This is called the frequency response method [14]. However, the calibration of transient electromagnetic receiving systems is not a one-time thing. The frequency response method not only requires a high-precision signal generator, but also must ensure the uniformity of the calibration magnetic field. It has high requirements for the field source and poor versatility, and cannot achieve on-site calibration of the device [9]. Reference [12] proposed a time-domain receiving coil parameter calibration method. By collecting the zero-state response of the receiving coil, the parameter solution is converted into a nonlinear programming problem. The parameters are solved by fitting the curve. The corresponding fitting effect will affect the accuracy of the parameter solution, and the sampling frequency is recommended to be above 1 MHz. Reference [9] proposed a time-domain feedback calibration method for coil sensors. The solution error of the induced electromotive force of the receiving coil is used as the feedback signal, and the exponential decay current is used as the calibration signal. The solution error of the induced electromotive force is extracted and used as the feedback signal. The calibration file is calibrated by reducing the non-steady-state interval of the feedback signal, eliminating the dependence on a uniform calibration magnetic field. However, the accuracy is lower than that in the literature [12], but fast calibration can be achieved.

To address the limitations of coil parameter shifts and uniform calibrated magnetic fields for field operations, this paper proposes a method for system parameter identification and online calibration of the transition process based on the response characteristics of the receiving coil system. It can realize the accurate calculation of the transfer function of the receiving coil system without the need for a specific calibration device and eliminates the dependence on the input source. It has the advantages of good versatility and convenience. First, this paper analyzes the system response of the receiving coil and proposes a method to change the system response characteristics through electronic switches to put the system in a damped state. Then, the system response in the underdamped state is analyzed, and the mapping relationship between the system response characteristic parameters and the parameters of the receiving coil itself is established, which equates the identification of the system inductance and capacitance parameters to the problem of solving two nonlinear equations. In order to improve the accuracy of the receiving coil parameter identification, the methods of improving the matching resistance and the parallel state switching capacitor are compared, and the method of parallel state switching capacitor is selected from the perspective of reducing the resonance point. Finally, the Newton method is used for parameter identification, which can realize the accurate determination of the receiving coil system parameters and transfer functions, and provides an accurate, simple, and online identification method for the coil sensor signal calibration.

## 2. Dynamic Response Analysis of Receiving Coil System

**Figure 1:**
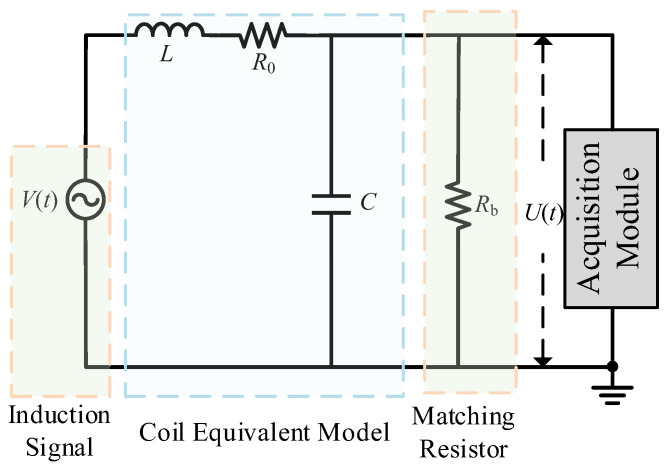
Receiving coil equivalent simplified circuit.

The equivalent circuit and signal acquisition diagram of the receiving coil are shown in Figure 1. *R*_0_, *L*, and *C* are the equivalent resistance, inductance, and distributed capacitance of the receiving coil; *R_b_* is the damping matching resistance of the receiving coil; *V*(*t*) is the induced electromotive force received by the receiving coil; and *U*(*t*) is the induced electromotive force measured by the acquisition module. When the receiving coil is collecting signals, the input and output system response functions are [15]

(1) $$H(s)=\frac{1}{LC[s^{2}+s(\frac{R_{0}}{L}+\frac{1}{R_{b}C})+\frac{R_{0}+R_{b}}{R_{b}LC}]}$$

The physical quantity natural frequency *ω*_n_ of this second-order system reflecting the frequency of its system vibration and the physical quantity damping ratio *ζ* reflecting the stability of the system and the dissipation characteristics of the system are, respectively,

(2) $$\omega_{n}=\sqrt{\frac{R_{b}+R_{0}}{R_{b}LC}}$$

(3) $$\zeta =\frac{R_{b}R_{0}C+L}{2\sqrt{R_{b}LC(R_{b}+R_{0})}}$$

When the receiving coil induces a unit impulse response *h*(*t*), the time domain expression of *H*(*s*) under different damping conditions is

(4) $$u(t)=\left\{\begin{array}{cc} \frac{R_{b}}{R_{0}+R_{b}}\left\{1-e^{-\delta t}\left[\cosh(\omega_{d}t)+\frac{\delta }{\omega_{d}}\sinh(\omega_{d}t)\right]\right\}, & \xi >1\\ \frac{R_{b}}{R_{0}+R_{b}}\left[1-e^{-\delta t}\left(1+\delta t\right)\right], & \xi =1\\ \frac{R_{b}}{R_{0}+R_{b}}\left\{1-e^{-\delta t}\left[\cos(\omega_{d}t)+\frac{\delta }{\omega_{d}}\sin(\omega_{d}t)\right]\right\}, & \xi <1 \end{array}\right.$$

(5) $$\delta =\xi \omega_{n}=\frac{1}{2}\left(\frac{R}{L}+\frac{1}{R_{b}C}\right)$$

where *δ* is the attenuation constant. According to Equation (4), after the induced signal passes through the receiving coil, the signal will inevitably change due to the influence of the transfer function. In order to accurately measure the magnetic field to be measured through the output signal of the coil, it is necessary to establish a mapping between the coil-induced electromotive force and the output signal. The induced electromotive force *U*(*t*) observed by the instrument is the time-domain convolution of the electromagnetic response signal *V*(*t*) and the transfer function *h*(*t*). Therefore, the mutual conversion between *U*(*t*) and *V*(*t*) can be achieved through deconvolution transformation [16,17].

(6) $$u(t)=v(t)*h(t)=F^{-1}\left[V(\omega )\times H(\omega )\right]$$

(7) $$v(t)=L^{-1}(V(s))=L^{-1}\left(\frac{Lu(t)}{H(s)}\right)$$

## 3. Receiving Coil Parameter Identification Method

### 3.1. Characteristic Analysis of Underdamped Oscillation

**Figure 2:**
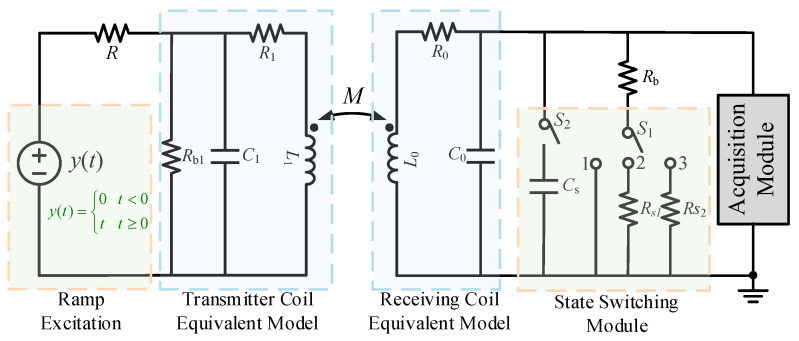
Schematic diagram of receiving coil parameter identification circuit.

In order to realize the parameter identification of the receiving coil, a parameter identification circuit is established, as shown in Figure 2. During normal operation, the state switching switches *S*_1_ and *S*_2_ are disconnected. In order to minimize the output signal distortion and obtain a high-quality output response and a relatively flat frequency response, matching resistors can be connected at both ends of the coil to put the system in a critical damping state. The calculation formula for the matching resistor at critical damping is

(8) $$R_{b}=\frac{L}{R_{0}C\pm 2\sqrt{LC}}$$

In practical applications, the matching resistance is usually appropriately reduced to make the system work in a slightly overdamped state to avoid oscillation caused by the system becoming underdamped due to uncertain factors.

**Figure 3:**
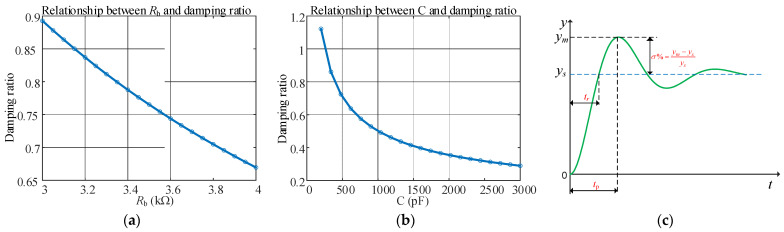
Dynamic response analysis of second-order system. (**a**) Capacitance *R*_b_ and damping ratio curve, (**b**) capacitance *C* and damping ratio curve, and (**c**) step response and characteristic parameters under underdamping.

As shown in Figure 3a,b, when calibrating the system, the system can be placed in an underdamped state by adding matching resistors and parallel state switching capacitors, and the parallel matching capacitor method has a greater impact on the damping coefficient.

According to the law of electromagnetic induction, when a ramp input signal is applied to the excitation coil (see Equation (9)), a step signal *ε*(*t*) will be induced in the receiving coil (see Equation (10)).

(9) $$y(t)=\left\{\begin{array}{c} 0\\ kt \end{array}\right.\begin{array}{c} t<0\\ t\geq 0 \end{array}$$

(10) $$\varepsilon (t)=\frac{d\Phi }{dt}\propto \frac{dy(t)}{dt}=kt$$

On the basis of reducing the damping coefficient by the above two methods, the system will change from the original slightly overdamped state to the underdamped oscillation state. From Figure 3c, the expressions of the adjacent peak time ∆*t_p_* and overshoot *σ%* of the underdamped step response are as follows:

(11) $$\Delta t_{p}=\frac{\pi }{\omega_{n}\sqrt{1-\zeta^{2}}}$$

(12) $$\sigma \%=\frac{y_{m}-y_{s}}{y_{s}}=e^{\left(-\frac{\zeta \pi }{\sqrt{1-\zeta^{2}}}\right)}\times 100\%$$

(13) $$y_{s}=\frac{KR_{b}}{R_{0}+R_{b}}$$

where *y_m_* is the system response amplitude, *y_s_* is the steady-state value of the system response, and *K* is the steady-state value of the signal.

### 3.2. Extraction and Calculation of Receiving Coil Characteristic Parameters

It can be seen from (13) that the steady-state value of the received signal is related to the steady-state value *K* of the induced signal, the matching resistor *R_b_*, and the coil parameter *R*_0_. Since the value of *R_b_* is generally much larger than that of *R*_0_, when *R*_0_ is obtained through the steady-state value, the identification error will increase due to the small proportion of *R*_0_. Therefore, the state switching switch *S*_1_ can be used to connect *R*_1_, *R*_2_, and the damping resistor *R_b_* in parallel to adjust the size of the matching resistor. At the same time, the unknown parameter *K* can be eliminated by comparing the two sets of results to remove the error of the excitation source signal size on the parameter identification. The corresponding calculation formula is as follows:

(14) $$\begin{array}{cc} R_{bn}=\frac{R_{b}*R_{n}}{R_{b}+R_{n}} & n=1,2 \end{array}$$

(15) $$R_{0}=\frac{(y_{s1}/y_{s2}-1)R_{b1}R_{b2}}{\left(R_{b1}-y_{s1}/y_{s2}*R_{b2}\right)}$$

**Figure 4:**
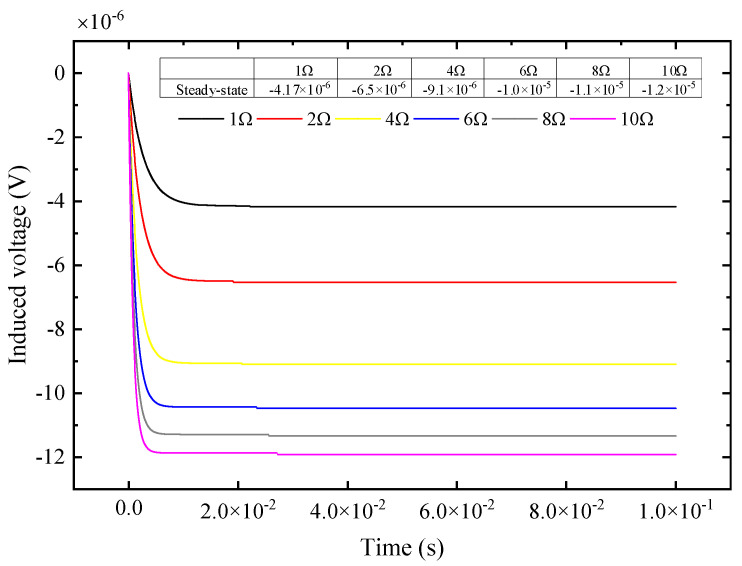
The induced voltage across the matching resistor under different resistances.

According to Equation (13), through the switch control of Figure 2, after setting switch *S*_1_ to 1 and 2, respectively, the different system steady-state values corresponding to the response signal are extracted. On the basis of the known two sets of matching resistance values and steady-state values, the *R*_0_ parameter can be obtained. The induced voltages corresponding to the two ends of different matching resistors are shown in Figure 4 below. The internal resistance of the receiving coil obtained by combining Equations (14) and (15) is shown in Table 1; the accurate measurement of coil *R*_0_ can be achieved by combining Equations (14) and (15) under simulation analysis.

It can be seen from Equations (11) and (12) that the two characteristic parameters ∆*t*_p_ and *σ*% are only related to the natural frequency and the damping ratio. After obtaining the underdamped pulse response curve of the second-order system, *y*_m_, *y*_s_ and the adjacent peak time ∆*t*_p_ can be extracted. Through the mapping relationship between this parameter and the natural frequency *ω*n and the damping ratio *ζ*, the natural frequency *ω*n and the damping ratio *ζ* can be accurately solved.

**Figure 5:**
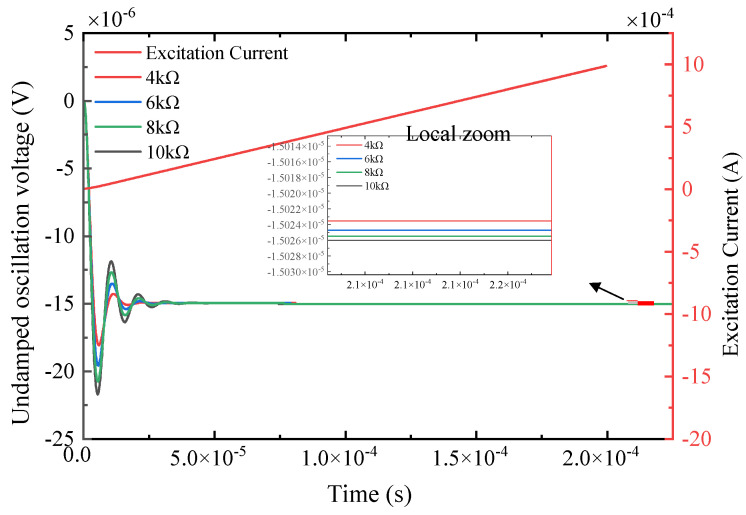
The induced voltage of the receiving coil and the excitation current of the transmitting coil under different matching resistances.

**Figure 6:**
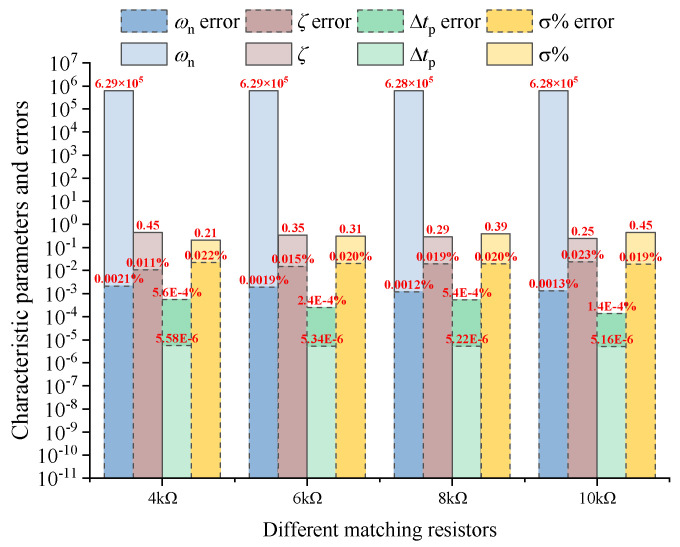
Characteristic parameters and errors under different matching resistances.

From Figure 3a, the receiving coil system can be placed in an underdamped state by increasing the matching resistance and switching the capacitor in parallel. First, we discuss the method of increasing the matching resistance: for the actual coil parameters shown in Table 1, different resistors are connected in series on the basis of the matching resistance. The underdamped oscillation response signal of the receiving coil when the electronic switch switches different matching resistances is as follows:

According to the response voltage signal shown in Figure 5, the characteristic parameters of the underdamped oscillation obtained by feature extraction are shown in the Figure 6.

As can be seen from Figure 6, when the method of increasing the matching resistance to make the system in underdamped oscillation is adopted, the absolute error of the values extracted from each parameter is small, but the value of the natural frequency itself is large. Under this absolute numerical error, the corresponding relative error is also large, and the parameter calculation result of the natural frequency will introduce a large error to the solution of the subsequent nonlinear equation.

**Figure 7:**
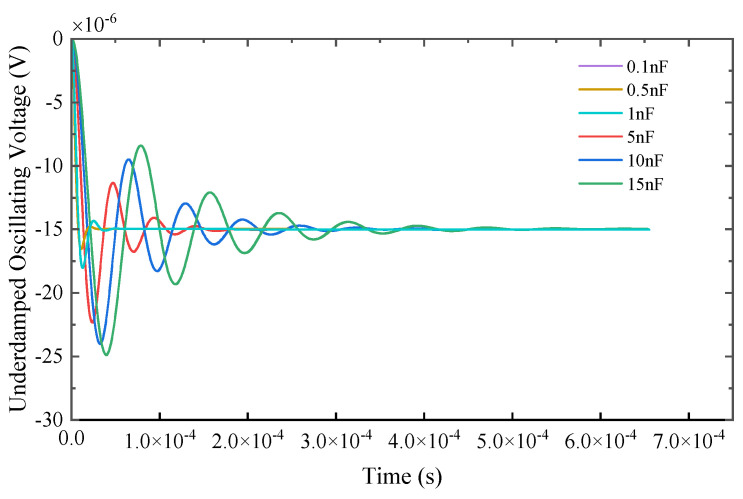
Underdamped response signals under different states of switched capacitors.

Based on the analysis that increasing the parallel resistance will introduce a large natural frequency calculation error, the response signal, characteristic parameters, and errors obtained by using the parallel state switching capacitor are shown in Figure 7.

It can be seen from Figure 7 that by changing the state of the switching capacitor, the damping coefficient of the receiving coil is significantly reduced, the system oscillation performance is enhanced, and the overshoot and peak time corresponding to the response signal are significantly improved.

**Figure 8:**
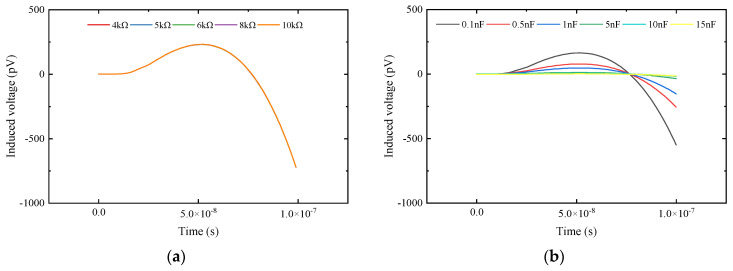
Comparison of early signals between the two methods. (**a**) Early signals under different matching resistances; (**b**) early signals under different states of switching capacitance.

As can be seen from Figure 8, due to the existence of the distributed capacitance of the receiving coil itself, as shown in (16), the receiving coil system has a resonant frequency, and the high resonant frequency of the coil itself will introduce oscillation in the early stage of the signal. A positive overshoot will appear in the early stage of the receiving coil, which will introduce inevitable errors in the feature extraction of the response signal.

(16) $$f=\frac{1}{2\pi \sqrt{LC}}$$

**Figure 9:**
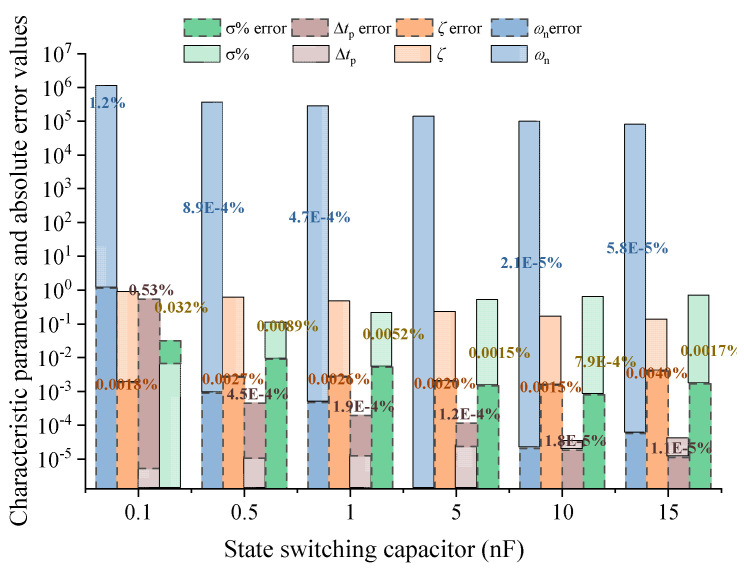
Characteristic parameters and errors of capacitors under different switching states.

As can be seen from Figure 8a, the overshoot size under different matching resistances is the same. As can be seen from Figure 8b, the method of parallel state switching capacitors can significantly reduce the corresponding resonant frequency point of the system, thereby reducing the error of early signal distortion on system parameter extraction. Comparing Figure 6 and Figure 9, it can be seen that, compared with the method of increasing matching resistance, the errors obtained by the method of parallel state switching capacitors are much smaller than those of the latter. Therefore, from the perspective of suppressing early signal oscillation and reducing signal extraction and calculation errors, this paper adopts the method of switch-controlled state switching capacitors to change the response characteristics of the receiving coil system and perform parameter identification.

**Figure 10:**
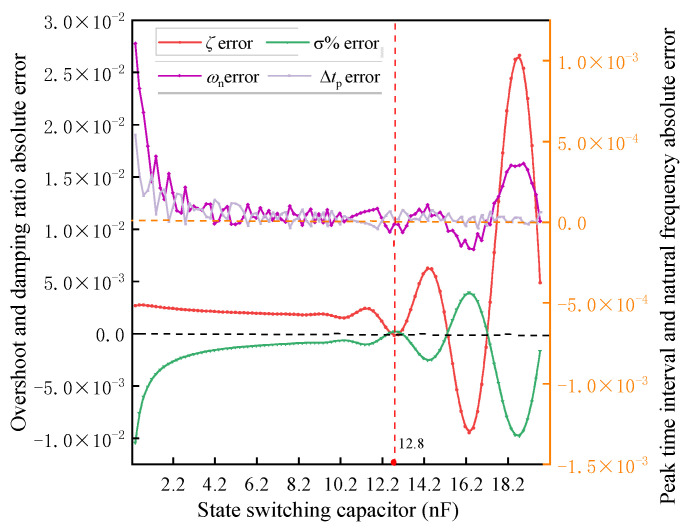
Relationship between state switching capacitance and characteristic parameter error.

For the parameters shown in Table 2 in this paper, the relationship between characteristic parameter extraction, calculation, and error under different state switching capacitors is analyzed, and the error curves obtained under 0.4 nF to 20 nF are shown in Figure 10.

From the characteristic parameter error relationship under different state switching capacitances in Figure 10, it can be seen that appropriately increasing the value of the state switching capacitance can reduce the parameter identification and calculation errors, but very large distributed capacitance will also lead to an increase in the error. Under the corresponding parameters in Table 2, the state switching capacitance value is taken as 12.8 nF.

### 3.3. Nonlinear Identification of System Parameters Based on Newton’s Method

For nonlinear equations, Newton’s method is a commonly used numerical method for solving the roots of nonlinear equations f(x) = 0, and it is also one of the most famous and effective methods. At this time, the double nonlinear equation system is

(17) $$F=\left[\begin{array}{c} f_{1}\\ f_{2} \end{array}\right]=\left[\begin{array}{c} \omega_{n}-\sqrt{\frac{R_{b}+R_{0}}{R_{b}LC}}\\ \zeta -\frac{R_{b}R_{0}C+L}{2\sqrt{R_{b}LC(R_{b}+R_{0})}} \end{array}\right]$$

Parameter iteration formula:

(18) $$x^{\left(k\right)}=x^{\left(k-1\right)}-J{\left(x^{\left(k-1\right)}\right)}^{-1}F(x^{\left(k-1\right)})$$

The function iteration process starts with selecting x(0). When calibrating the pulsed eddy current receiving coil signal, the initial parameters of the receiving coil are known, and the parameters to be identified are offset based on the initial values. The offset is small. Therefore, the initial value based on Newton’s method is close to the true value, which can significantly improve the convergence speed and reduce the number of iterations.

**Figure 11:**
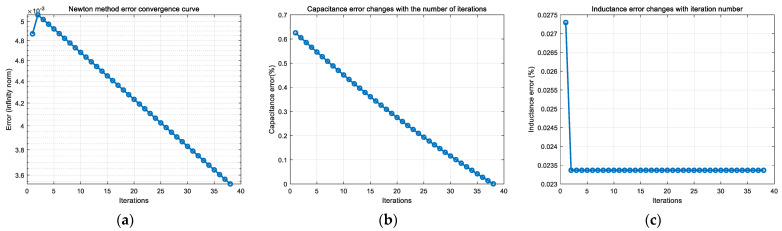
Parameter identification results based on Newton’s method. (**a**) Newton’s method convergence curve from experimental test, (**b**) error curve of capacitance parameters with iteration number, and (**c**) error curve of inductance parameters with iteration number.

On the basis of accurately extracting and calculating the characteristic parameters of the underdamped response signal of the receiving coil, the state switching capacitor is set to 12.8 nF, and the initial value of the receiving coil is as shown in Table 1. The Newton iteration method is applied, and the convergence curve and capacitance and inductance identification errors are shown in Figure 11.

**Figure 12:**
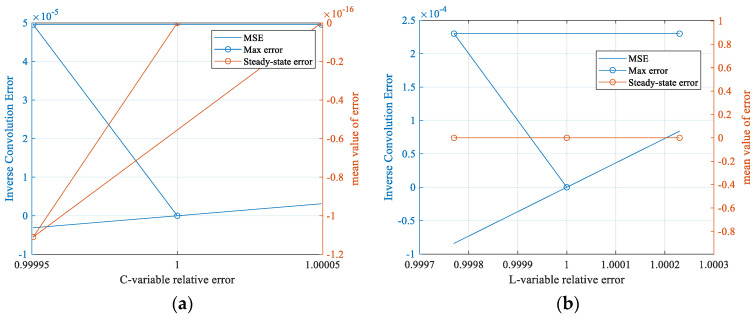
Inverse convolution signal accuracy analysis with solving accuracy. (**a**) Analysis of deconvolution results with capacitance parameter solving accuracy; (**b**) analysis of inverse convolution results with inductive parameter solving accuracy.

From Figure 11, it can be seen that, after signal extraction and feature identification, and then solved by nonlinear equations, the absolute error of the inductance of the receiving coil can be up to 0.023%, and the absolute error of the capacitance can be up to 0.004956%, which possesses a similar accuracy compared with the preset parameter deviations of *L* and *C* in the literature [9] that ultimately converge to 0.00447% and 0.00454%, respectively, but the method is more convenient, which can realize the accurate identification of receiving coil parameters. In order to analyze the effect of the error values of the coil parameters on the deconvolution accuracy of the response signal at this solution accuracy, the steady state error, the maximum error, and the mean score error between the deconvolved signal waveform and the real signal of the response signal at this accuracy obtained by the solution are shown in Figure 12.

As can be seen from Figure 12, the accuracy of the waveforms obtained from the acquired response signals after deconvolution is high at this solution accuracy, with a maximum error point error of 4.956 × 10^−5^ for the waveform due to capacitance, and 2.3 × 10^−4^ for the waveform due to inductance, and the waveforms are almost identical at this solution accuracy.

## 4. Experimental Results

**Figure 13:**
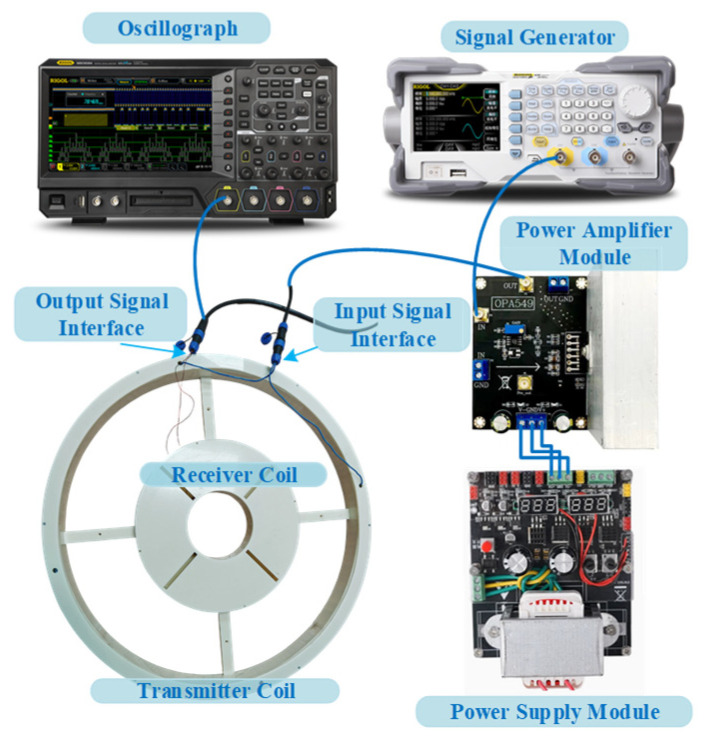
Schematic diagram of experimental test.

**Figure 14:**
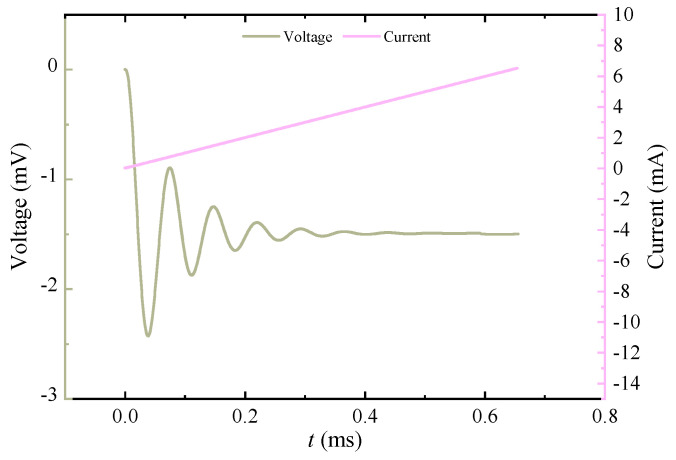
Applied excitation current and induced voltage signal.

In order to verify the accuracy of the receiving system parameter identification and signal calibration method based on underdamped oscillation, the excitation source generates a standard ramp voltage signal through the RIGOL DG1000Z series signal generator, and then the signal is linearly amplified by the power amplifier module and applied to the excitation coil; the pulsed eddy current ground detection coil adopts a central loop structure, the excitation coil diameter is 0.8 m, and the receiving coil diameter is 0.2 m. A schematic diagram of the experimental test is shown in Figure 13. The excitation and response signals are shown in Figure 14.

**Figure 15:**
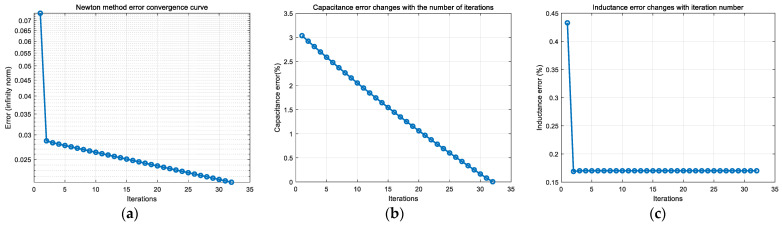
Capacitor and inductor identification errors under experimental tests. (**a**) Newton’s method convergence curve from experimental test, (**b**) error curve of capacitance parameters with iteration number, and (**c**) error curve of inductance parameters with iteration number.

The characteristic parameters obtained by signal extraction and calculation are shown in Table 3. The corresponding inductance and capacitance error curves obtained by signal parameter identification using this characteristic parameter are shown in Figure 15.

As can be seen from Figure 15, by applying a step signal to the excitation coil, collecting the underdamped response curve of the receiving coil, and extracting and calculating the characteristic parameters of the response curve, the parameter identification based on the Newton method can achieve accurate and rapid identification of the receiving coil parameters. Among them, under experimental conditions, the inductance identification error of the receiving coil can reach 0.17%, and the minimum error of the capacitance parameter can reach 0.038%. The introduction of interference signals leads to the acquisition of the signal in the parametric extraction, and the parameters’ extracted error becomes larger. In the subsequent nonlinear equation solving, the iterative process is based on the premise of the extracted parameters for the exact value of the parameter solution, so the extraction of the parameter perturbation will inevitably lead to parameter solving accuracy reduction.

## 5. Conclusions

In the actual application of pulsed eddy current receiving coil, the parameters will be offset and affect the measurement accuracy. In the conventional measurement process using an impedance analyzer, there are inconveniences. This paper proposes a receiving system parameter identification method based on the following underdamped dynamic response characteristics:

(1) The methods of increasing matching resistance and parallel capacitance to change the system state are compared. From the perspective of feature extraction and calculation accuracy of response signal characteristic parameters, the parallel capacitance method is proved to be better.
(2) On the basis of obtaining the accuracy characteristic parameters, the Newton method is used. After parameter identification, the absolute error of the inductance of the receiving coil can be reduced to 0.023%, and the absolute error of the capacitance can be reduced to 0.004956%.
(3) Under experimental conditions, the inductance identification error of the receiving coil can reach 0.17%, and the minimum error of the capacitance parameter can reach 0.038%. The parameter identification method proposed in this paper can efficiently, accurately, and conveniently identify and calibrate the system parameters of the receiving coil online, which is conducive to improving the signal inversion progress and detection accuracy in the pulsed eddy current detection process.

## Figures and Tables

**Table 1:** Coil *R*_0_ calculated under different parallel resistances.

Resistance (Ω)	1	2	4	6	8	10
*R*_0_ (Ω)	2.6	2.6	2.6	2.6	2.6	2.6

**Table 2:** Receiving coil parameters.

Receiving Coil Parameters	*L* (mH)	*C* (pF)	*R* (Ω)
Initial value	10	250	2.6
Real value	10.09	251.6225	2.6

**Table 3:** Signal extraction and calculation results.

Characteristic Parameters	Specific Values
Peak	2.4631 mV
Adjacent peak time	0.0364 ms
Steady-state value	1.5017 mV
Overshoot	64.03%

## Data Availability

The data used in the analysis presented in this paper will be made available subject to the approval of the data owner.
